# Frequent reduced expression of alpha-1B-adrenergic receptor caused by aberrant promoter methylation in gastric cancers

**DOI:** 10.1038/sj.bjc.6603555

**Published:** 2007-01-23

**Authors:** H Noda, Y Miyaji, A Nakanishi, F Konishi, Y Miki

**Affiliations:** 1 Department of Molecular Diagnosis, Japanese Foundation for Cancer Research, 3-10-6, Ariake, Koto-ku, Tokyo 135-8550, Japan; 2 Department of Surgery, Omiya Medical Center, Jichi Medical School, Omiya, Japan; 3 Medical Research Institute, Tokyo Medical and Dental University, Tokyo, Japan

**Keywords:** *ADRA1B*, gastric cancer, intestinal metaplasia, DNA methylation

## Abstract

Recent studies have suggested that epigenetic inactivation of tumour-related genes by promoter methylation participates in the development of gastric cancer. We newly identified the frequently aberrant promoter methylation of *alpha-1B-adrenergic receptor* (*ADRA1B*) in colorectal cancer by methylation-sensitive representational difference analysis (MS-RDA) and examined the methylation status of the *ADRA1B* promoter in 34 paired samples of colorectal cancer and surrounding epithelial tissue, and 34 paired samples of gastric cancer and surrounding epithelial tissue. In colorectal cancers, only four of 34 (11.8%) tumours showed *ADRA1B* promoter methylation. In contrast, *ADRA1B* promoter methylation was detected in 24 of 34 (70.6%) gastric cancers and in 14 of 34 (41.2%) surrounding epithelial tissues. The frequency of *ADRA1B* promoter methylation was higher in gastric epithelial tissues with intestinal metaplasia (41.6%) than in those without intestinal metaplasia (25.0%). Reverse transcription–PCR detected reduced ADRA1B expression in 12 of 18 (66.7%) gastric cancers, and its promoter methylation was detected in 11 of these 12 (91.7%) gastric cancers with reduced ADRA1B expression. Thus, *ADRA1B* promoter is frequently methylated in gastric cancer. Our results suggest that the *ADRA1B* gene is an important tumour-related gene frequently involved in the development and progression of gastric cancer.

Aberrant DNA methylation is a feature of human cancers, characterised by generalised hypomethylation and regional hypermethylation (Gama-Sosa *et al*, 1983; Feinberg *et al*, 1988). When regional hypermethylation occurs in CpG sites within the promoter region of tumour-suppressor genes or tumour-related genes that normally are unmethylated, gene transcription is inhibited, similar to the effects of mutations and deletions (Herman *et al*, 1994; Merlo *et al*, 1995; Baylin and Herman, 2000; Esteller *et al*, 2001). There is a growing list of tumour-suppressor genes or tumour-related genes associated with CpG island methylation in cancers (Leung *et al*, 1999; Kang *et al*, 2000; Shim *et al*, 2000; Kaneda *et al*, 2002a, 2002b; Li *et al*, 2002; Byun *et al*, 2003; Chan *et al*, 2003; Tokumaru *et al*, 2003; Choi *et al*, 2004; Miotto *et al*, 2004).

Gastric cancer is the leading cause of cancer-related mortality in Japan and many other countries (Fuchs and Mayer, 1995). Although the molecular genetics of gastric cancer remain unclear, accumulating evidence suggests that many tumour-suppressor genes and tumour-related genes are inactivated by promoter methylation (Leung *et al*, 1999; Kang *et al*, 2000; Shim *et al*, 2000; Kaneda *et al*, 2002a, 2002b; Li *et al*, 2002; Byun *et al*, 2003; Chan *et al*, 2003; Tokumaru *et al*, 2003; Choi *et al*, 2004; Miotto *et al*, 2004). Among various genes, methylation in the CpG island of the *hMLH1* gene, which encodes for the DNA mismatch repair protein MLH1, has been linked to a substantial proportion of sporadic gastric cancers with microsatellite instability (Fleisher *et al*, 1999; Leung *et al*, 1999). Some gastric cancers are characterised by a high degree of concordant methylation of CpG islands, including *p16, E-cadherin*, and *hMLH1*; such tumours are classified as high CpG island methylator phenotype (CIMP) (Toyota *et al*, 1999). Epigenetic inactivation of tumour-related genes by promoter methylation thus seems to have an important role in the development of gastric cancer.

To enable a genome-wide search for differences in CpG methylation between cancer and normal tissue, methylation-sensitive representational difference analysis (MS-RDA) was developed by Ushijima *et al* (1997). This method demonstrated reduced expression of the *INSIG1* gene and possible silencing of the *p41Arc* gene due to promoter methylation in gastric cancer (Kaneda *et al*, 2002a). In addition, several genes were also shown to be inactivated by aberrant promoter methylation in human cancers (Takai *et al*, 2001; Kaneda *et al*, 2002b; Asada *et al*, 2003). In this study, we used the MS-RDA technique to analyse two human colorectal cancers and newly identified *alpha-1B-adrenergic receptor* (*ADRA1B*). In colorectal cancers, only a subset of tumours showed aberrant *ADRA1B* promoter methylation. In contrast, *ADRA1B* promoter methylation was found much more frequently not only in gastric cancers but also in their surrounding epithelial tissues, and the majority of gastric cancers with *ADRA1B* promoter methylation had reduced ADRA1B expression. Our results suggest that aberrant *ADRA1B* promoter methylation with a consequent reduction in ADRA1B expression may be involved in gastric carcinogenesis.

## MATERIALS AND METHODS

### Clinical materials

Thirty-four paired samples of colorectal cancer and surrounding epithelial tissue, and 34 paired samples of gastric cancer and surrounding epithelial tissue were obtained at the time of surgery with informed consent. In addition, three samples of gastric epithelial tissue free of gastric cancer were obtained from the patients who underwent pancreaticoduodenectomy for the treatment of pancreatic cancer. Samples were immediately frozen in liquid nitrogen and stored at −80°C until DNA and RNA extraction. Among the 34 samples of surrounding gastric epithelial tissue, intestinal metaplasia (IM) was found in 26 (76.5%) on histopathological examination.

### Mmethylation-sensitive representational difference analysis, sequencing, and database search

Methylation-sensitive representational difference analysis was performed as described by Ushijima *et al* (1997), using DNA obtained from two paired samples of colorectal cancer and surrounding epithelial tissue. Briefly, genomic DNAs of cancer and surrounding epithelial tissue were digested by *Hpa*II (New England Biolabs, Beverly, MA, USA), and the Rhpa adaptor was ligated to the digest. *Hpa*II-amplicon was prepared by PCR. The Rhpa adaptor of the *Hpa*II-amplicon from the corresponding sample of normal tissue was removed by *Msp*I digestion, gel-purified (Gel Extraction Kit; Qiagen, Hilden, Germany), and switched to JHpa adaptor. The *Hpa*II-amplicon from the surrounding epithelial tissue was mixed with an excess amount of that from cancer tissue to perform competitive hybridisation, followed by PCR with JHpa primer. After two cycles of competitive hybridisation, the products were cloned into pGEM-T Easy vector (Promega, Madison, WI, USA). Then, plasmid DNA was cycle sequenced with the SP6 and T7 primers, using a CEQ Dye Terminator Cycle Sequencing Quick Start Kit (Beckman Coulter, Inc., Fullerton, CA, USA), and a CEQ2000XL DNA analyser (Beckman Coulter, Inc.). Homology searches were performed with BLAST program at the GenBank web site.

### Methylation-specific PCR for *ADRA1B* promoter in colorectal and gastric cancers and surrounding epithelial tissues

We performed methylation-specific PCR (MSP) to determine the methylation status of *ADRA1B* promoter in 34 paired samples of colorectal cancer and surrounding epithelial tissue and 34 paired samples of gastric cancer and surrounding epithelial tissue, using bisulphite-modified genomic DNA as described by Herman *et al* (1996). In brief, 1 *μ*g of DNA was denatured by NaOH and modified by sodium bisulphite. The DNA sample was then purified with Wizard DNA purification resin (Promega Corp.), treated again with NaOH, ethanol precipitated, and resuspended in H_2_O. We used four primer sets (Region 1, nucleotides −590 to −506; Region 2, nucleotides −517 to −274; Region 3, nucleotides −323 to −213; and Region 4, nucleotides −225 to −61) to comprehensively investigate the methylation status of *ADRA1B* promoter (nucleotides −754 to +173) (Ramarao *et al*, 1992). Because Region 2 includes a very wide area, which contains many CpGs, we set MSP for Region 3, which extensively overlaps with the 3′ end of the Region 2. The transcription start site was defined as +1, and the primer sets and PCR conditions are described in Table 1. Human genomic DNA treated *in vitro* with *SssI* methylase (New England Biolabs, Inc, Beverly, MA, USA) was used as positive control. The PCR products were analysed on 2% agarose gels with ethidium bromide and visualised under UV illumination. The presence of a visible PCR product in sets for methylated specific DNA was judged to be methylation-positive.

### Bisulphite sequencing of *ADRA1B* promoter in gastric cancers and surrounding epithelial tissues

We performed bisulphite sequencing of *ADRA1B* promoter in 10 randomly selected paired samples of gastric cancer and surrounding epithelial tissue. Bisulphite-modified DNA was used for PCR with primers common for methylated and unmethylated DNA sequences, which amplified a product containing 68 CpG sites (nucleotides −672 to −59) in *ADRA1B* promoter. The primer sets and PCR conditions are described in Table 2. The PCR products were gel-purified (Gel Extraction Kit; Qiagen, Hilden, Germany) and were cloned into pGEM-T Easy vector (Promega). Eight recombinants were cycle sequenced with the SP6 and T7 primers, using a CEQ Dye Terminator Cycle Sequencing Quick Start Kit and a CEQ2000XL DNA analyser (both from Beckman Coulter, Inc.). The methylation status of each CpG site was determined by sequencing, as unmethylated cytosines are converted into thymines by bisulphite treatment, whereas methylated cytosines remain unaltered.

### Semiquantitative reverse transcription(RT)–PCR

Total RNA was prepared from 18 paired samples of gastric cancer and surrounding epithelial tissue for which the methylation status of *ADRA1B* promoter had been assessed by MSP. The total RNA was immediately treated with DNase I (Life Technologies, Rockville, MD, USA) and reverse-transcribed using a Superscript III reverse transcriptase kit (Life Technologies) to prepare first-strand cDNA. A *β-actin* fragment was amplified as an internal control. The primer set and PCR conditions are described in Table 3.

### 5q loss of heterozygosity analysis

5q loss of heterozygosity (LOH) analysis was carried out using a single-nucleotide polymorphism (SNP) in the *ADRA1B* gene (5q23–q32), three SNPs in the *adenomatous polyposis coli* gene (5q21–q22), and an SNP in the *interferon regulator factor-1* gene (5q31.1) for the 18 paired samples of gastric cancer and surrounding epithelial tissue examined by RT–PCR. Detailed information about these five SNPs is available from JSNP (http://snp.ims.u-tokyo.ac.jp). Sequence change in SNP from the PCR product of surrounding epithelial tissue to that from the cancer tissue was judged as 5q LOH positive. The primer sets and PCR conditions are described Table 4; the primers for PCR were used as sequence primers.

### Statistical analysis

The Fishers' exact test and Student's *t*-test were used to examine associations between two categorical variables. The level of statistical significance was set at *P*<0.05.

## RESULTS

### Isolation of DNA fragments aberrantly methylated in colorectal cancer

DNAs from two paired samples of colorectal cancer and surrounding epithelial tissue were used as tester and driver for MS-RDA. We obtained 33 DNA fragments by MS-RDA, and the genomic origins of the 33 DNA fragments were analyssed by sequencing and a GenBank database search. One DNA fragment matched the promoter of the *ADRA1B* gene, which is located in 5q23–q32.

### *ADRA1B* promoter methylation in colorectal and gastric cancers and surrounding epithelial tissues

**Figure 1:**
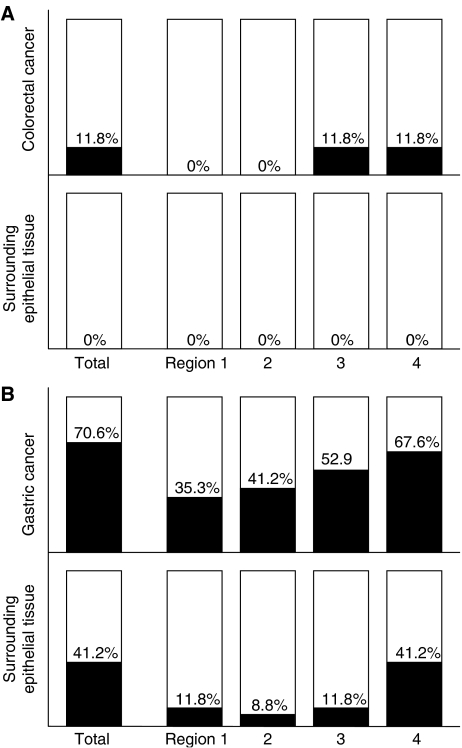
*Alpha-1B-adrenergic receptor* promoter methylation was detected in four of 34 (11.8%) colorectal cancers and was not detected in surrounding colorectal epithelial tissues. *Alpha-1B-adrenergic receptor* promoter methylation was thus considered a cancer-specific event in colorectal cancers and surrounding epithelial tissues. In contrast, *ADRA1B* promoter methylation was detected in 24 of 34 (70.6%) gastric cancers and in 14 of 34 (41.2%) surrounding epithelial tissues. In gastric cancers, the frequency of *ADRA1B* promoter methylation increased progressively from Region 1 to Region 4. In surrounding epithelial tissues, the frequency of *ADRA1B* promoter methylation was also highest in Region 4.

As shown in Figure 1A, *ADRA1B* promoter methylation was detected by MSP in four of 34 (11.8%) colorectal cancers. In all four of these colorectal cancers, methylation was limited to Region 3 and Region 4 in *ADRA1B* promoter. In contrast, no *ADRA1B* promoter methylation was detected in the 34 corresponding samples of normal colorectal epithelial tissue. Thus, *ADRA1B* promoter methylation was a cancer-specific event in the colorectal cancers and surrounding epithelial tissues.

As shown in Figure 1B, *ADRA1B* promoter methylation was detected in 24 of 34 (70.6%) gastric cancers and in 14 of 34 (41.2%) surrounding epithelial tissues. *Alpha-1B-adrenergic receptor* promoter methylation in gastric cancers and in surround epithelial tissues showed no significant correlation with clinicopathological characteristics such as age and gender of the patients or the location, stage, and differentiation of their tumours. *Alpha-1B-adrenergic receptor* promoter methylation was not detected in the three samples of gastric epithelial tissue obtained from patients with pancreatic cancer unassociated with gastric cancer. Among the 34 gastric cancers, *ADRA1B* promoter methylation was detected in Region 1 in 12 cancers (35.3%), Region 2 in 14 (41.2%), Region 3 in 18 (52.9%), and Region 4 in 23 (67.6%). Therefore, in gastric cancers, the frequencies of *ADRA1B* promoter methylation increased progressively from Region 1 to Region 4. Among the 34 surrounding epithelial tissues, *ADRA1B* promoter methylation was detected in Region 1 in four samples (11.8%), Region 2 in three (8.8%), Region 3 in four (11.8%), and Region 4 in 14 (41.2%). For each region, the frequency of *ADRA1B* promoter methylation was lower in the surrounding epithelial tissues than in the cancers; however, similar to the cancers, the frequency of *ADRA1B* promoter methylation was highest in Region 4 in the surrounding epithelial tissues.

**Figure 2:**
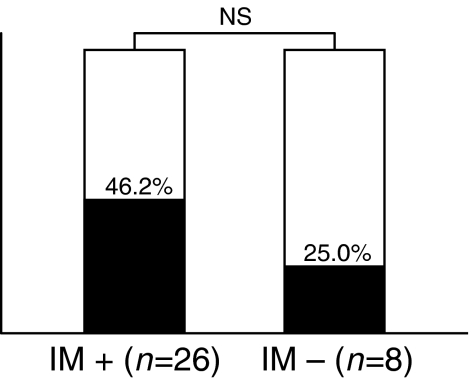
The frequency of *ADRA1B* promoter methylation in gastric epithelial tissues with IM (41.6%) was slightly but not significantly higher than that in gastric epithelial tissues without IM (25.0%).

When we analysed the correlations between *ADRA1B* promoter methylation and the presence of IM in surrounding epithelial tissues, *ADRA1B* promoter methylation was detected in 12 of 26 (46.2%) gastric epithelial tissues with IM and in two of eight (25.0%) gastric epithelial tissues without IM (Figure 2). The frequency of *ADRA1B* promoter methylation was slightly but not significantly higher in gastric epithelial tissues with IM than in gastric epithelial tissues without IM.

**Figure 3:**
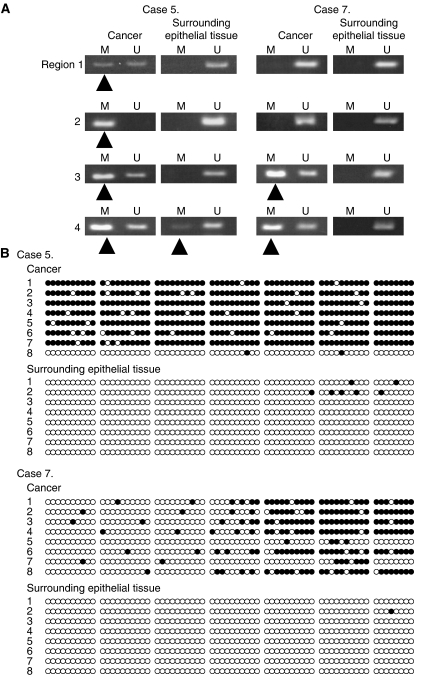
Methylation status of *ADRA1B* promoter was analysed by methylation-specific PCR (MSP) and bisulphite sequencing of 68 CpG sites in eight clones obtained by PCR of bisulphite-modified DNA. (**A**) Representative results of MSP for two paired samples of gastric cancer and surrounding epithelial tissue. The presence of a visible PCR product in the lanes marked M indicates the presence of methylated alleles (black arrow). (**B**) Schemas of the results of bisulphite sequencing of the same samples as those analysed by MSP. Unmethylated and methylated cytosines are shown by open and closed circles, respectively. In Case 5, analysis of DNA from cancer tissue showed *ADRA1B* promoter methylation in Regions 1, 2, 3, and 4 on MSP and widespread methylation in seven of eight clones on bisulphite sequencing. In Case 7, analysis of DNA from cancer tissue showed *ADRA1B* promoter methylation in Regions 3 and 4 on MSP; methylation in these regions was also revealed by bisulphite-sequencing in six of eight clones. These two cases of cancer also had reduced ADRA1B expression. In Case 7, analysis of DNA from the surrounding epithelial tissues showed no *ADRA1B* promoter methylation in any region on MSP; similar results were obtained on bisulphite sequencing. In Case 5, analysis of DNA from the surrounding epithelial tissues showed methylation in Region 4 on MSP, but no methylation in any clone on bisulphite sequencing. In these two cases, the surrounding epithelial tissues showed no reduced ADRA1B expression. Although DNA was judged to be methylation-positive in the surrounding epithelial tissues of Case 5, the amount of methylated DNA was probably much less than that of unmethylated DNA, resulting in no apparent methylation on bisulphite sequencing.

In 10 analysed gastric cancers, the results of bisulphite sequencing of *ADRA1B* promoter methylation were concordant with those of MSP regarding the extent of the methylation area (Figure 3A and B). Four of 10 samples judged to be methylation-negative on MSP showed sequence changes of nearly all cytosines to thymines in eight recombinants in the *ADRA1B* promoter on bisulphite sequencing. Six of 10 samples judged to be methylation-positive on MSP exhibited methylation in nearly all eight recombinants in the same region. Seven of 10 analysed samples of surrounding epithelial tissue judged to be methylation-negative on MSP showed sequence changes of nearly all cytosines to thymines in eight recombinants in the *ADRA1B* promoter on bisulphite sequencing, similar to Case 7. In three of 10 samples of surrounding epithelial tissues judged to be methylation-positive in Region 4 on MSP, bisulphite sequencing showed sequence changes of nearly all cytosines to thymines in eight recombinants in the *ADRA1B* promoter, similar to Case 5. In most gastric cancers positive for *ADRA1B* promoter methylation, the band of PCR products obtained with sets for methylated specific DNA was more intense than that obtained with sets for unmethylated specific DNA, similar to Case 5 and Case 7 (Figure 3A). In some of the corresponding epithelial tissues positive for *ADRA1B* promoter methylation, the band of PCR products obtained with sets for methylated specific DNA was less intense than that obtained with sets for unmethylated specific DNA, similar to Case 5 (Figure 3A). Therefore, even in samples judged to be methylation-positive, the quantity of methylated DNA was less than that of unmethylated DNA in the surrounding epithelial tissues, and this difference may account for the inconsistency between the results of MSP and bisulphite sequencing for the surrounding epithelial tissues.

### Correlation among reduced ADRA1B expression, *ADRA1B* promoter hypermethylation, and 5q LOH in gastric cancers and surrounding epithelial tissues

**Figure 4:**
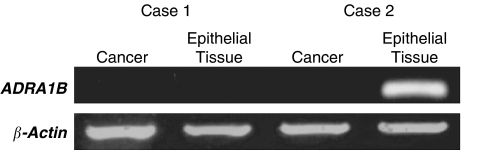
ADRA1B expression in gastric cancers was examined by RT–PCR. In this series, samples of cancer tissue from Case 1 and Case 2 and samples of surrounding epithelial tissue from Case 1 had markedly reduced ADRA1B expression as compared with that of the surrounding epithelial tissue of Case 2. As shown in Table 5, the samples of cancer tissue from Case 1 and Case 2 and of surrounding epithelial tissue from Case 1 exhibited *ADRA1B* promoter methylation in Region 2 and/or Region 3. No *ADRA1B* promoter methylation was found in the surrounding epithelial tissue of Case 2.

Semiquantitative RT–PCR detected reduced ADRA1B expression in 12 of 18 (66.6%) gastric cancers and three of 18 (16.4%) surrounding tissues (Figure 4). Table 5 shows correlations among *ADRA1B* promoter methylation status according to region, 5q LOH, and ADRA1B expression in 18 paired samples of gastric cancer and surrounding epithelial tissue. All three cases with reduced ADRA1B expression in surrounding epithelial tissue also had reduced ADRA1B expression in their corresponding cancers. Eleven of 12 (91.7%) gastric cancers and three of three (100.0%) corresponding tissues with reduced ADRA1B expression also had promoter methylation in Region 2 and/or Region 3, and three of 11 (27.3%) gastric cancers with reduced ADRA1B expression also had 5q LOH. On the other hand, three gastric cancers and six surrounding epithelial tissues with *ADRA1B* promoter methylation did not have reduced ADRA1B expression. In most of these tissue samples, *ADRA1B* promoter methylation was limited to Region 1 and/or Region 4, and no methylation was found in either Region 2 or Region 3. Thus, although one of 12 (8.3%) gastric cancers with reduced ADRA1B expression had no *ADRA1B* promoter methylation or 5q LOH, most cases with reduced ADRA1B expression had promoter methylation and 5q LOH; methylation in Region 2 and Region 3 strongly correlated with inactivation of ADRA1B expression.

## DISCUSSION

We applied the MS-RDA method to two paired samples of colorectal cancer and surrounding epithelial tissue, and identified *ADRA1B.* The *ADRA1B* promoter was aberrantly methylated in a subset of colorectal cancers, but not in corresponding epithelial tissues, suggesting that such methylation is a cancer-specific event during colorectal carcinogenesis. Furthermore, in gastric cancers, the *ADRA1B* promoter was very frequently methylated, generally in association with reduced ADRA1B expression. *Alpha1-adrenergic receptors* are members of the superfamily of G protein-coupled receptors and mediate effects related to the regulation of cellular hypertrophy and proliferation (Cruise *et al*, 1985; Lefkowitz and Caron, 1988; Cotecchia *et al*, 1990; Thonberg *et al*, 1994; Spector *et al*, 1997). Three distinct subtypes of *alpha1-adrenergic receptors* (*alpha1A-* (*ADRA1A*)*, alpha1B-* (*ADRA1B*)*, and alpha1D-* (*ADRA1D*) *adrenergic receptors*) have a prominent role in cell growth, and activation of *ADRA1A* and *ADRA1B* inhibits serum-prompted cell proliferation (Auer *et al*, 1998; Shibata *et al*, 2003). *ADRA1B* can activate the cyclin-dependent kinase inhibitors *p27KIP1* and *p21Cip1/WAF1*, thereby inhibiting cell proliferation through this pathway (Auer *et al*, 1998; Shibata *et al*, 2003). Reduced ADRA1B expression might thus disrupt this pathway, giving cells aberrant growth advantage.

Our results first demonstrated *ADRA1B* promoter methylation in colorectal and gastric cancers. *Alpha-1B-adrenergic receptor* promoter methylation was particularly frequent in gastric cancers, whereas it was infrequent in colorectal cancers. Kim *et al* (2004) reported that 60% of gastric cancer cell lines and 64% of gastric cancers were methylated at the *RUNX3* promoter, while *RUNX3* promoter methylation was detected in only 4.9% colorectal cancers. Thus, the frequency of promoter methylation of a given tumour suppressor gene appears to vary among different types of cancer. Our results suggest that *ADRA1B* promoter methylation plays an important role in gastric cancer, but not in colorectal cancer, similar to *RUNX3* promoter methylation. Furthermore, RT–PCR detected reduced ADRA1B expression in 12 of 18 (66.7%) gastric cancers, 11 (91.7%) of which concurrently had *ADRA1B* promoter methylation in Region 2 and/or Region 3. These results clearly suggested that *ADRA1B* promoter methylation is the principal mechanism for gene inactivation, with methylation of Region 2 and Region 3 (−517 to −213 relative to the transcription start site) in the promoter being especially critical. Three of 11 (27.3%) gastric cancers with reduced ADRA1B expression also had 5q LOH. Therefore, 5q LOH is apparently related to reduced ADRA1B expression in a subset of gastric cancers.

We also demonstrated that *ADRA1B* promoter methylation occurred in the surrounding epithelial tissues of gastric cancers, a small fraction of which concurrently had reduced ADRA1B expression. Several studies have shown that promoter methylation of multiple tumour-related genes is present in gastric epithelial tissues with or without cancer, and that accumulations of such genes promote gastric carcinogenesis (Leung *et al*, 1999; Kang *et al*, 2000; Kang *et al*, 2001, 2003a, 2003b; To *et al*, 2002; Waki *et al*, 2002; Chan *et al*, 2003). Waki *et al* (2002) reported that among 94 samples obtained from patients with gastric cancer, promoter methylation of *E-cadherin, hMLH1*, and *p16* was found in 67, 24, and 44% of the surrounding gastric epithelial tissues, respectively. Kang *et al* (2003a) reported that among 268 samples obtained from gastric epithelial tissues without cancer, promoter methylation of *DAP-kinase, E-cadherin, THBS1, TIMP3, p14, MGMT, p16, COX2, GSTP1 hMLH1,* and *RASSF1A* was observed in 53.7, 41.0, 37.7, 23.1, 18.7, 10.9, 10.0, 4.1, 3.4, 1.7, and 0.4%, respectively. Our results demonstrated that *ADRA1B* promoter methylation was present in 14 of 34 (41.2%) surrounding epithelial tissues of gastric cancers; this frequency was similar to those of the important tumour-related genes mentioned above. Kang *et al* (2003b) also demonstrated that the average number of methylated genes markedly increases from non-neoplastic mucosa to intestinal-type gastritis. Although still controversial, the precancerous nature of IM has been suggested by the clinical phenomenon that gastric cancer often arises from intestinal-type gastritis. Although the difference did not reach statistical significance, the frequency of *ADRA1B* promoter methylation in surrounding epithelial tissues with IM was higher than that in surrounding epithelial tissues without IM. Thus, *ADRA1B* promoter methylation may also participate in the early phase of gastric carcinogenesis, similar to the other tumour-related genes mentioned above (Stemmermann, 1994). The degree of promoter methylation and the frequency of reduced ADRA1B expression in cancer are more extensive and more frequent than those in the surrounding epithelial tissues. With gastric carcinogenesis, *ADRA1B* promoter methylation spreads extensively, leading to reduced ADRA1B expression. Such reduced expression gives cell aberrant growth potential, resulting from loss of the growth inhibitory activity of ADRA1B.

In conclusion, our study showed that *ADRA1B* promoter is aberrantly hypermethylated in colorectal and gastric cancers. In gastric cancer, *ADRA1B* promoter methylation occurs frequently in both cancer tissue as well as in surrounding epithelial tissue. Our results suggest that *ADRA1B* is an important tumour-related gene with key roles in the development and progression of gastric cancer.

## Figures and Tables

**Table 1:** Primer sets and PCR conditions of methylation-specific PCR for *ADRA1B* promoter

**Sense primer**	**Antisense primer**	**Amplicon size (bp)**	**Annealing temperature (°C)**	**Cycles**
*Region 1*				
(M) GGGTGATTCGCGATTTTTAC	CTCCCAAATCACCTCTACGA	83	56	40
(U) GTGGGTGATTTGTGATTTTTATGT	CTCCCAAAATCACCTCTACAAA	85	56	40
				
*Region 2*				
(M) CGTTTAAGGTTCGTTTTCGC	AAAAAAATCTACTTCAATAAACCGCT	243	56	40
(U) TTATGTTTAAGGTTTGTTTTTGTGG	AAAAAATCTACTTCAATAAACCACT	245	54	40
				
*Region 3*				
(M) TGGATTCGTATTGTTTTTTAGTGTC	AAAAAATCTACTTCAATAAACCGCT	110	58	35
(U) TTGGATTTGTATTGTTTTTTAGTGTGTTG	AAAAAATCTACTTCAATAAACCACT	111	58	35
				
*Region 4*				
(M) AAGTAGATTTTTTTCGGCGTTC	AACTCCAAATTTAATAATCCACGTC	165	60	35
(U) AGTAGATTTTTTTTGGTGTTTGT	AACTCCAAATTTAATAATCCACATC	165	60	35

(M)=methylated DNA specific; (U)=unmethylated DNA specific; *ADRIB*=*alpha-1B-adrenergic receptor*; PCR=polymerase chain reaction.

**Table 2:** Primer set and PCR conditions of bisulphite sequencing for *ADRA1B* promoter

**Sense primer**	**Antisense primer**	**Amplicon size (bp)**	**Annealing temperature (°C)**	**Cycles**
TATTAAAGGTAAGTAGTTTTTAATTTATTT	ACAACTCCAAATTTAATAATCCAC	614	58	40

*ADRA1B*=*alpha-1B-adrenergic receptor*; PCR=polymerase chain reaction.

**Table 3:** Primer set and PCR conditions of RT–PCR for ADRA1B expression

**Sense primer**	**Antisense primer**	**Amplicon size (bp)**	**Annealing temperature (°C)**	**Cycles**
GCTAAGACGTTGGGCATTGT	GTTGAAGTAGCCCAGCCAGA	144	60	35

*ADRA1B*=*alpha-1B-adrenergic receptor*; PCR=polymerase chain reaction; RT=reverse transcription.

**Table 4:** Primer sets and PCR conditions for the region including SNP in *ADRA1B, APC*, and *IRF-1*

**Sense primer**	**Antisense primer**	**Amplicon size (bp)**	**Annealing temperature (°C)**	**Cycles**
***ADRA1B*** (JSNP ID; IMS-JST087433: A4154019G)				
CTGGTCACGCGGAGGAAG	GGTTCTTGGTGGTTCTCTTTGG	244	60	35
				
***APC*** (JSNP ID; IMS-JST061883: C113263G)				
TGCTTGAAAATTCCAGTGTCA	GGACATTTTTGACCGCAGTT	382 bp	62	35
				
***APC*** (JSNP ID; IMS-JST041076: A14531742C, and JSNP ID; IMS-JSTO41075: G14531786A)				
GCCAGGATATGGAAAAACGA	TTCCAAGGCAGAACAGAACA	255 bp	62	35
				
***IRF-1*** (JSNP ID; IMS-JST005685: T34238200C)				
ATCAGCAGCCAGAGGGTAGA	CTGGCAAAAGCATCTGTGAA	231 bp	62	35

*ADRA1B*=*alpha-1B-adrenergic receptor*; PCR=polymerase chain reaction.

**Table 5:** Correlation among *ADRA1B* promoter methylation^a^, 5q LOH, and ADRA1B

**Cancer**							**Surrounding epithelial tissue**					
**Case region**	**1**	**2**	**3**	**4**	**Expression**	**5q LOH**	**Case region**	**1**	**2**	**3**	**4**	**Expression**
1	M	U	M	M	Down	Negative	1	U	U	M	M	Down
2	U	M	M	U	Down	Negative	2	U	U	U	U	Normal
3	U	U	U	M	Normal	Negative	3	U	U	U	M	Normal
4	U	U	U	U	Normal	Negative	4	U	U	U	U	Normal
5	M	M	M	M	Down	Negative	5	U	U	U	M	Normal
6	U	M	U	U	Down	Positive	6	U	U	U	U	Normal
7	U	U	M	M	Down	Negative	7	U	U	U	U	Normal
8	U	U	U	M	Normal	Negative	8	U	U	U	U	Normal
9	M	M	M	M	Down	Negative	9	M	M	M	M	Down
10	U	U	U	U	Normal	Negative	10	U	U	U	U	Normal
11	U	U	U	U	Normal	Negative	11	U	U	U	U	Normal
12	U	M	M	M	Down	Negative	12	U	U	U	M	Normal
13	M	M	M	M	Down	Negative	13	U	M	M	M	Down
14	U	U	U	M	Normal	Negative	14	U	U	U	U	Normal
15	U	U	U	U	Normal	Negative	15	U	U	U	U	Normal
16	M	M	M	U	Down	Positive	16	U	U	U	U	Normal
17	U	U	M	M	Down	Positive	17	U	U	U	U	Normal
18	M	U	U	M	Normal	Negative	18	U	U	M	M	Normal

Expression in 18 paired samples of gastric cancer and surrounding epithelial tissue.

a Methylation status of the *ADRA1B* promoter in each region is represented as M or U. M indicates methylation-positive, and U indicates methylation-negative (unmethylated).

*ADRA1B*=*alpha-1B-adrenergic receptor*; LOH=loss of heterozygosity.
